# Mesenchymal Stem Cells as Immunomodulators in a Vascularized Composite Allotransplantation

**DOI:** 10.1155/2012/854846

**Published:** 2012-11-25

**Authors:** Yur-Ren Kuo, Chien-Chang Chen, Shigeru Goto, Pao-Yuan Lin, Fu-Chan Wei, Chao-Long Chen

**Affiliations:** ^1^Center for Vascularized Composite Allotransplantation, Kaohsiung Chang Gung Memorial Hospital and College of Medicine, Chang Gung University, Kaohsiung 833, Taiwan; ^2^Department of Plastic and Reconstructive Surgery, Kaohsiung Chang Gung Memorial Hospital and College of Medicine, Chang Gung University, Kaohsiung 833, Taiwan; ^3^Liver Transplantation Program and Department of Surgery, Kaohsiung Chang Gung Memorial Hospital and College of Medicine, Chang Gung University, Kaohsiung 833, Taiwan

## Abstract

Vascularized composite allotransplantations (VCAs) are not routinely performed for tissue reconstruction because of the potentially harmful adverse effects associated with lifelong administration of immunosuppressive agents. Researchers have been eagerly seeking alternative methods that circumvent the long-term use of immunosuppressants. Mesenchymal stem cells (MSCs) show promise as an immunomodulatory therapeutic agent and are currently being tested in preclinical and clinical settings as therapies for autoimmune disorders or transplant rejection. The mechanisms by which MSCs modulate the immune response are still under thorough investigation, but these most likely involve expression of local factors influencing T-cell regulation, modulation of cytokine expression (e.g., IL-10, TGF-**β**, TNF-*α*, INF-**γ**, etc.), and interactions with dendritic or antigen presenting cells. In this paper, we summarize the current understanding of immunomodulation achieved by MSC therapies and introduce a possible outline for future clinical applications in VCA.

## 1. Introduction

Avascularized composite allotransplantation (VCA) consists of various tissue combinations, including muscle, nerve, tendon, skin, bone, cartilage, and bone marrow. VCA serves as an ideal solution for the replacement or repair of certain tissues following traumatic loss, tumor resection, or repair of congenital abnormalities [1]. Recently, important advances have been made and new studies demonstrate that VCA is clinically feasible [2, 3]. Indeed, 78 successful human hand and 14 partial face allotransplantations have been performed [4–6]. However, VCA is not routinely performed for tissue repair and reconstruction because lifelong administration of immunosuppressive agents, which have potentially harmful side effects, is necessary to avoid rejection of the highly antigenic skin tissue [7–9]. Furthermore, even if patient compliance is excellent, conventional immunosuppressive protocols may not be sufficient to prevent chronic rejection [4, 10, 11]. Consequently, researchers have been eagerly seeking alternative methods of establishing lifelong tolerance while minimizing toxicity. Studies have reported numerous active clinical trials in which mesenchymal stem cells (MSCs) are used in the treatment of inflammatory diseases, such as graft-versus-host disease (GVHD) [12, 13], Crohn's disease [14, 15], ulcerative colitis [16], multiple sclerosis [17, 18], and systemic lupus erythematosus [19, 20]. Herein, we focus on the immunomodulatory effects of MSCs, provide a snapshot of the results from current *in vitro* and *in vivo* studies, and discuss future prospects in which these procedures can be made widely available in VCA. 

## 2. Mesenchymal Stem Cells

Mesenchymal stem cells (MSCs), which originate in the bone marrow, are multipotential nonhematopoietic progenitor cells capable of differentiating into various mesenchymal cell types. Bone marrow (BM) stromal cells were first identified by Friedenstein, who described an adherent fibroblast-like population, which was able to differentiate into bone, that he referred to as osteogenic precursor cells [21]. Subsequent studies demonstrated that these cells have the ability to differentiate into various other mesodermal cell lineages, including chondrocytes, osteocytes, tenocytes, and myoblasts, and this ability is currently used as a functional criterion in defining MSCs [22, 23]. Recent studies have identified pluripotent cells that not only differentiate into cells of the mesodermal lineage, but also into endodermal and neuroectodermal lineages, including neurons, hepatocytes, and endothelial cells [24]. Based on this multilineage differentiation capacity, Caplan introduced the term mesenchymal stem cells (MSCs) [25].

### 2.1. Source and Characteristics of MSCs

**Figure 1:**
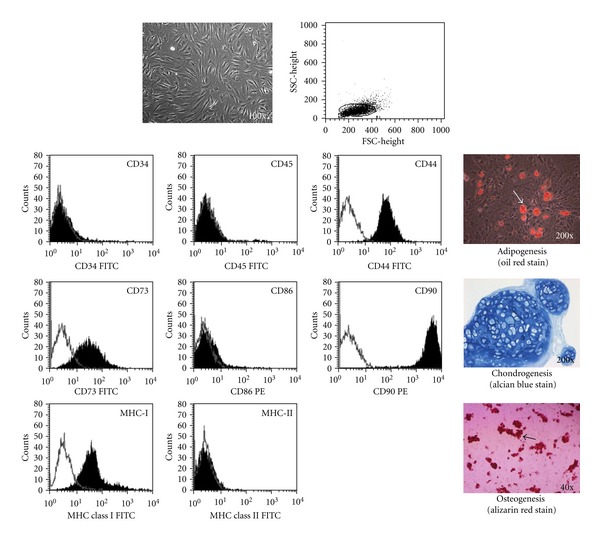
The phenotypic characterization and differentiation potential of mesenchymal stem cells (MSCs) *in vitro*. Mesenchymal stem cells were expanded in culture and demonstrated positive surface staining for CD44, CD73, CD90, and MHC class I, but not for CD34, CD45, MHC class II, and CD86 expression, as detected by flow cytometry. MSCs were further tested for their ability to differentiate into adipocytes, osteoblasts, and chondrocytes. Osteoblasts were identified by Alizarin red staining, lipid droplets were identified by oil-red O staining, and chondrogenic differentiation was visualized by Alcian blue staining.

Although MSCs were originally isolated from BM, similar pluripotent cell types have been isolated from other tissues, including adipose tissue, placenta, amniotic fluid, and fetal tissues, such as lung [22, 26, 27]. They can also be isolated from cord blood, synovial tissue and, at extremely low frequencies, from adult peripheral blood [26, 28]. Currently, no specific marker or combination of markers have been identified that specifically defines MSCs. MSCs have been expanded in culture, *ex vivo*, and have been phenotypically characterized by flow cytometry. MSCs expressed CD44, CD73, CD90, MHC class I, CD105, and CD166, as determined by positive surface staining. MSCs are devoid of hematopoietic and endothelial markers such as MHC class II, CD11b, CD14, CD31, CD34, CD45, and CD80/B7-1 [26–28]. The capacity to differentiate into multiple mesenchymal lineages, including adipocytes, osteoblasts, and chondrocytes, is used as a functional criterion to define MSCs (Figure 1).

### 2.2. MSC-Mediated Immunosuppression *In  Vitro*

#### 2.2.1. The Interaction between MSCs and T Cells

Previous studies have revealed that MSCs do not express immunogenic costimulatory molecules such as B7-1, B7-2, or CD40. Therefore, it is likely that they are unable to stimulate alloreactive T cells [29, 30]. Glennie et al. suggested that bone marrow MSCs can arrest the division of activated T cells and induce T-cell anergy. Studies have demonstrated that MSCs can suppress T-lymphocyte activation and proliferation *in vitro*, and that this inhibition affects the proliferation of T cells induced by alloantigens and mitogens, as well as the activation of T cells by CD3 and CD28 antibodies [31]. Further study has indicated that soluble factors are involved, as the separation of MSCs and peripheral blood mononuclear cells (PBMCs) by a semipermeable transwell membrane does not prevent inhibition of proliferation [32]. Supernatants from human and mouse MSC cultures show no inhibitory effect unless MSCs have been cocultured with lymphocytes, suggesting that the suppressive factor(s) are not constitutively secreted by MSCs, but require dynamic cross-talk between MSCs and T lymphocytes [33].

Our previous study tested the effects of co-cultured adipose-derived mesenchymal stem cells (ASCs) and allogeneic T cells from a completely MHC-mismatched strain. BrdU proliferation assays have revealed a statistically significant reduction in the proliferation of T cells that were co-cultured with ASCs, compared to T cells that were cultured alone [34]. Our study further revealed a statistically significant reduction in the proliferation of T cells that were co-cultured with syngeneic MSCs [35]. These data indicate that the suppression of T-cell proliferation by MSCs has no immunological restriction, as similar suppressive effects were observed with cells that were autologous or allogeneic to the responder cells. 

#### 2.2.2. MSC-Induced T-Cell Regulation

It is commonly accepted that immunosuppression can be accomplished by lymphocyte populations that are known as regulatory T cells. The regulatory T-cell population resides mainly within the CD4+ T-cell subset; specifically, these cells are described as CD4+CD25+ forkhead box P3+ (Foxp3+) (Treg) cells [36, 37]. CD4+CD25+ regulatory T cells have emerged as a unique population of T cells that help to maintain a peripheral immune tolerance [38, 39]. In our study, we analyzed *in vitro* the percentage of CD4+/CD25+/Foxp3+ regulatory T cells and revealed that this population was significantly increased in MSC and T cell co-cultures, as compared to T cells cultured alone [34]. These results indicate that MSCs both suppressed T-cell proliferation and increased the number of regulatory T cells.

The mechanism by which the anti-proliferative effects of MSCs are delivered has not yet been elucidated, although several candidate molecules have been proposed [30, 40, 41]. Previous studies have indicated that MSCs actively inhibit the functions of several immune cells through enzymatic activity and the secretion of cytokines and growth factors [40–42]. The mechanisms underlying these effects are not fully understood, but they appear to involve both cell contact and a range of soluble factors, including transforming growth factor (TGF)-*β*, interleukin-10 (IL-10), interferon-*γ* (IFN-*γ*), metabolites of tryptophan that are generated by the activation of indoleamine-2,3-dioxygenase (IDO), or prostaglandin E2 (PGE2) [33, 43–46]. Recent studies have elucidated an important and complex role for TGF-*β* in regulatory T-cell biology [44, 47]. The disruption of TGF-*β* signaling in T cells impairs the maintenance of regulatory T cells, which results in the expansion of activated effector T-cell populations [48]. Aggarwal and Pittenger showed that coculturing MSCs with differentiated effector T cells simultaneously led to a decreased release of the proinflammatory cytokine IFN-*γ* from Th1 cells, an increase in IL-4 release from Th2 cells, and an increase in the proportion of regulatory T cells [42]. These data provide a strong evidence that MSCs can induce a shift from a pro-inflammatory to an anti-inflammatory state.

#### 2.2.3. MSCs Inherent Maturation Process of Dendritic Cells (DC)

Dendritic cells (DCs) play a key role in the induction of immunity and tolerance, depending on their activation and maturation stages and, as recently proposed, the cytokine milieu at the sites of inflammation [49, 50]. Mature DCs express high levels of MHC class II, CD80, and CD86, which are well described in antigen presentation to CD4+ T cells [51]. Thus, DC maturation plays a key role in initiating T cell responses to evade immunity. DCs have the ability to initiate a primary adaptive immune response through the capture, processing, and presentation of antigen to naive CD4+ T cells; however, differences in the capacities of DCs to initiate these responses are linked to the developmental maturation state of the DC [52]. Our study revealed that recipient immature DC pulsed alloantigen combined with a short-term immunosuppressant could significantly increase hind-limb allograft survival in rodents and could increase the percentage of regulatory cells* in vivo* [42, 53]. MSCs have been demonstrated to interfere with the differentiation and maturation of DCs by suppression of the expression of MHC class II, CD80, and CD86 [54, 55]. These data indicated that MSCs can modulate DC maturation and decrease T-cell activation. 

## 3. Immunomodulatory Effects of MSCs in VCA 

### 3.1. MSCs Suppress GVHD in a VCA Model

It has been previously demonstrated that the combination of bone marrow transplantation (BMT) and immunosuppressant administration prolongs organ transplant survival [56]. Despite the promising potential of mixed allogeneic chimerism in the induction of VCA tolerance, graft-versus-host disease (GVHD), secondary to the introduction of donor BMT, and toxicity from ablative host conditioning are considered to be the main hurdles in the widespread acceptance of this technique [57, 58]. Studies have indicated that donor MSCs are potent inhibitors of T-cell proliferation in mixed lymphocyte cultures, thus preventing GVHD caused by total-body-irradiation-(TBI-) BMT and prolonging skin allograft survival in rodent models [59, 60]. Pan and colleagues have indicated a potential use of MSCs for the induction of stable and high level mixed hematopoietic chimerism and subsequent donor specific tolerance in a rat hind-limb VCA under a nonmyeloablative conditioning regimen [61]. In our study, we used a swine heterotopic hind-limb VCA model under TBI as a nonmyeloablative conditioning regimen [62]. The results revealed that TBI, combined with BMT and short-term cyclosporine-A (CsA) treatment, induced GVHD-related symptoms [63]. However, multiple rounds of donor MSC therapy combined with BMT, after TBI and short-term CsA treatment, appear to modulate GVHD and prevent graft rejection [63].

### 3.2. MSCs Prolong VCA Survival 

The immunomodulatory effects and therapeutic potential of MSCs in organ transplantation have resulted in successful preclinical applications for composite tissue and organ allotransplantation (Table 1) [34, 35, 43, 53, 60, 61, 64–67]. We have investigated the effects of MSCs on prolonged VCA survival by measuring the immunosuppressive activity that was rendered by multiple injections of adipose-derived MSCs (ASCs, 2 × 10^6^/dose on days 7, 14, and 21 after transplantation), short-term anti-lymphocyte serum (ALS) and CsA in a rodent hind-limb model [34]. The results revealed that ALS-CsA-ASCs significantly prolonged VCA survival without rejection, as compared to the results observed in ALS-CsA and untreated control groups [34]. We adjusted the protocol to be applicable to a large animal VCA model, and our results demonstrated that the administration of multiple injections of donor MSC injections (2 × 10^7^/dose on days 7, 14, and 21 post-transplantation) without BMT, combined with preoperative irradiation and short-term CsA, has similar results on allotransplant survival in the swine hind-limb VCA model [35]. Our results led us to speculate that BMT is unnecessary to prolong VCA survival if MSCs are used as an immunosuppressant. To reconfirm this hypothesis and to test another VCA model for pre-clinical study, we applied MSCs in another large animal study, using a miniature swine hemifacial VCA model (consisting of skin paddle, muscle, ear cartilage, and lymphoid gland tissue) [68]. The difference between the hemi-facial VCA model and the hind-limb model is that the hemi-facial model does not contain donor vascularized bone, but does include more alloskin area and lymphoid gland tissue. Our results revealed that the MSC-CsA group had significantly prolonged hemi-facial VCA survival [66]. However, the survival of a VCA composite with vascularized bone in the hind-limb model is significantly longer than that of the hemi-facial model without vascularized bone. These results indicated that multiple injections of MSCs can prolong VCA survival, especially in a model of a hind-limb VCA composite with vascularized bone marrow. 

### 3.3. MSCs Modulate T-Cell Regulation in VCA 

To assess the regulation of T-cells in VCA that was treated with MSCs, regulatory-like T cells in circulating blood were detected by flow cytometry, and topical tissue expression of allotransplants was examined by immunohistochemical (IHC) staining in the rodent and miniature swine VCA models. In our rodent hind-limb VCA model, the flow cytometric analysis of recipient peripheral blood revealed that CD4+/CD25+/Foxp3+ regulatory T-cell populations were significantly increased at early time points (4–6 weeks after transplant) in the animals that were treated with multiple rounds of ASCs and short-term ALS-CsA, as compared to the ALS-CsA and control groups [34]. Furthermore, IHC staining of alloskin biopsies revealed significantly higher numbers of Foxp3+ T cells in the subcutaneous and dermis layers of skin from the animals that were treated with ASCs, CsA, and ALS, as compared to the other groups [34]. In our miniature swine hind-limb VCA study, flow cytometric analysis of recipient peripheral blood revealed that CD4+/CD25+ regulatory-like T-cell populations increased significantly in animals that were treated with MSCs, CsA, and irradiation at 2 weeks and 6 weeks following transplant, as compared to controls. Foxp3+ T cell populations increased significantly in the animals that were treated with MSCs, CsA, and irradiation at 2 weeks following transplant [35]. The percentages of regulatory-like T-cells gradually declined to a normal ratio by 300 days after transplant. In contrast, IHC staining of graft skin tissue biopsies revealed significant numbers of CD25+ T cells in the subcutaneous and dermis layers in animals treated with MSCs, CsA, and irradiation, as compared to the CsA alone and control groups [34, 35]. These results demonstrate that treatment with MSCs, combined with a short-term immunosuppressant regimen, increased the percentages of regulatory T-cell populations at the early time points after allotransplantation; however, this effect decreases with time.

### 3.4. MSCs Induce Mixed Chimerism in VCA

To evaluate the donor-specific chimerism in the rodent hind-limb VCA model (Brown-Norway (BN) to Lewis (LEW) strain), donor lymphoid cells (RT1^*n*^  (+) cells) from the peripheral blood of the long-term survivors were examined by flow cytometric analysis. Our study revealed a significant increase in the population of RT1n-expressing BN donor cells in circulating blood from the recipients (LEW) that were treated with MSCs and short-term immunosuppressant therapy [34]. This study demonstrates that donor MSCs upregulate donor-cell microchimerism in recipients. These hind-limb allotransplants contain vascularized bone-marrow, which provides a constant resource of donor-specific progenitor cells and stromal cells, where the latter are essential for the proliferation and differentiation of BM-derived cells into hematopoietic progenitors.

### 3.5. MSCs Modulate Cytokine Expression in VCA 

Studies have indicated that MSCs actively inhibit the functions of several immune cell types through enzymatic activity and the secretion of cytokines and growth factors such as TGF-*β*, IL-10, IFN-*γ*, and indoleamine-2,3-dioxygenase (IDO), PGE2 [44, 48, 69]. In our previous study, recipient peripheral blood serum was analyzed by ELISA following different treatments in a rodent hind-limb VCA model [34]. The data revealed that the circulating concentrations of both TGF-*β*l and IL-10, but not IFN-*γ*, increased significantly in VCA that were treated with multiple rounds of adipose-derived MSCs and short-term CsA-ALS at 4 weeks and 21 weeks following transplant, as compared to controls [34]. In our miniature swine hemi-facial VCA model, the concentrations of the soluble forms of TNF-*α*, IL-10, and TGF-*β*l were determined by ELISA following the various treatments [66]. An analysis of recipient peripheral blood serum revealed that TNF-*α*  levels were decreased significantly in the groups that were treated with MSCs alone or MSC-CsA at 2 weeks after transplantation, as compared to those in controls. Further analysis of recipient peripheral blood serum showed a trend toward increased TGF-*β*1 levels in MSC-treated groups, as compared to control groups. IL-10 levels were increased significantly in animals that were treated with MSC or MSC-CsA at 2 weeks after transplantation, as compared to controls [66].

In contrast, IHC staining of alloskin biopsies revealed significantly lower numbers of CD45 and IL-6 positive cells in the subcutaneous and dermis layers of skin from animals that were treated with MSC or MSC-CsA, compared to control animals [66]. The alloskin biopsies revealed a significant increase in the number of TGF-*β*l positive cells in the subcutaneous and dermis layers of skin from animals that were treated with MSC at 2 weeks after transplantation and MSC-CsA groups at 2 and 6 weeks following transplantation compared to controls [66]. These data indicate that regulatory-like T-cell subset-related cytokines were involved in MSC-induced immune tolerance and allotransplant survival. 

### 3.6. Homing of Exogenous MSCs in VCA

Homing is the process by which cells migrate to, and engraft in, the tissue in which they can exert local and functional effects. To explore the mechanisms by which MSCs modulate allograft survival, the homing of MSCs in VCA recipients was tracked using BrdU-labeled donor MSCs [63]. BrdU-labeled donor MSCs were intravenously injected into the recipient swine in our large animal VCA model, followed by an investigation of MSC homing and engraftment. Our data revealed a significant population of BrdU-labeled donor MSCs in the subcutaneous layers of both the donor and recipient skin and the perivascular parenchyma of the recipient liver, as detected by horseradish peroxidase-diaminobenzidine (HRP-DAB) staining [63]. This result indicated that the hematogenous spread of MSCs could enable engraftment and proliferation of these cells in the recipient tissue. 

## 4. Conclusion

**Figure 2:**
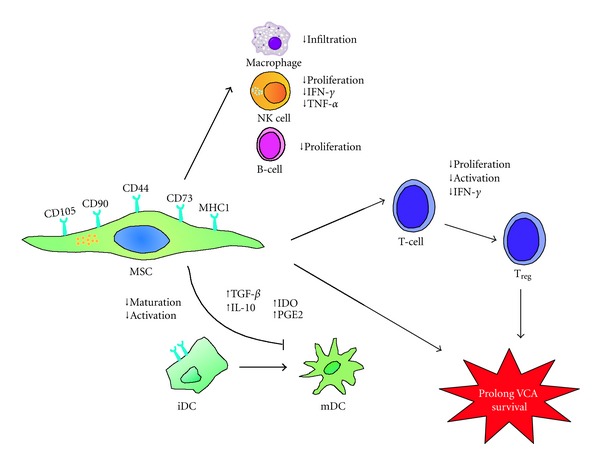
Proposed immunomodulatory mechanisms of MSCs in a vascularized composite allotransplantation (VCA). MSCs mediate their immunomodulatory effects by interacting with cells from both the innate (DCs, macrophages, NK cells) and adaptive immune (T cells and B cells) systems, particularly through the regulation of T-cell proliferation and the inhibition of DC differentiation. MSC inhibition of TNF-*α*  and IFN-*γ* secretion, promotion of IL-10 and TGF-*β* secretion, and IDO and PGE2 expression may affect the maturation states and functional properties of DCs, resulting in skewing of the immune response toward the prolongation of VCA survival. DC: dendritic cells; IFN-*γ*: interferon (IFN)-*γ*; IDO: indoleamine-2,3-dioxygenase; NK: natural killer; PGE2: prostaglandin E2; T_reg_: regulatory T-cells; TGF-*β*: transforming growth factor-*β*; TNF-*α*: tumor necrosis factor (TNF)-*α*.

The therapeutic potential of MSCs in VCA has recently generated great interest and enthusiasm. The effects of MSCs can be exploited to produce a potent immunosuppressive response, as virtually all immune cells are susceptible, and much is expected from the use of these cells in allotransplantation. Multiple infusions of MSCs, combined with a nonmyeloablative preconditioning regimen (e.g., irradiation, antilymphocyte serum) and transient immunosuppression, could effectively prevent GVHD and prolong VCA survival. This prolongation of survival might occur because MSCs promote the engraftment of donor progenitor cells and modulate the host immune function. Based on our previous results and other reports, we suggest that the biomechanisms of MSC suppression involve the modulation of cytokine expression (e.g., IL-10, TGF-*β*, TNF-*α*, INF-*γ*) and regulatory T-cell subsets, as we propose in Figure 2. Thus, MSC infusion is a potentially novel strategy for clinically improving VCA survival and inducing immune tolerance. 

## Figures and Tables

**Table 1:** Pre-clinical allotransplant models utilizing MSC for immune modulation.

Authors	Animal model	Allotransplant model	MSC source	Combined short-term immunosuppressant	Outcome
Kuo et al. (2009) [53]	Swine	Hind-limb VCA (allogeneic)	Donor bone marrow	CsA	MSC alone, prolong allograft survival; MSC + TBI + BMT + CsA, significantly prolong graft survival

Pan et al. (2010) [61]	Rat	Hind-limb VCA (allogeneic)	Allogeneic bone marrow	Rapamycin + ALG	MSC + rapamycin + ALG + TBI + BMT prolong allograft survival and induce mixed chimerism

Kuo et al. (2011) [35]	Swine	Hind-limb VCA (allogeneic)	Donor bone marrow	CsA	MSC + CsA + TBI prolong allograft survival

Kuo et al. (2011) [34]	Rat	Hind-limb VCA (allogeneic)	Allogeneic adipose tissue	ALS + CsA	MSC + ALS + CsA prolong allograft survival and induce immune tolerance

Kuo et al. (2012) [66]	Swine	Facial VCA (allogeneic)	Donor bone marrow	CsA	MSC + CsA prolong allograft survival

Itakura et al. (2007) [65]	Rat	Islet cell transplant (allogeneic)	Allogeneic bone marrow	CsA	MSC + BMT + CsA prolong islet allograft survival and induce immune tolerance

Kim et al. (2011) [43]	Rat	Islet cell transplant (allogeneic)	Autologous bone marrow	CsA	Prolong islet allograft survival

Casiraghi et al. (2008) [64]	Mouse	Heart (semiallogeneic)	Donor allogeneic bone marrow	—	Prolong heart allograft survival

Ge et al. (2009) [67]	Mouse	Heart (allogeneic)	Donor allogeneic bone marrow	Rapamycin	Prolong heart allograft survival

Sbano et al. (2008) [60]	Rat	Alloskin transplantation (allogeneic)	Donor allogeneic bone marrow	CsA	MSC + CsA prolong skin allograft survival
